# Association of West Nile virus illness and urban landscapes in Chicago and Detroit

**DOI:** 10.1186/1476-072X-6-10

**Published:** 2007-03-12

**Authors:** Marilyn O Ruiz, Edward D Walker, Erik S Foster, Linn D Haramis, Uriel D Kitron

**Affiliations:** 1 Department of Pathobiology, University of Illinois, 2001 South Lincoln Ave, Urbana, IL, USA; 2 Department of Microbiology & Molecular Genetics, Michigan State University, 2215 Biomed Phys Sci Bldg, East Lansing, MI, USA; 3 Michigan Department of Community Health, 201 Townsend St, Lansing, MI, USA; 4 Illinois Department of Public Health, 535 W. Jefferson St, Springfield, IL, USA

## Abstract

**Background:**

West Nile virus infection in humans in urban areas of the Midwestern United States has exhibited strong spatial clustering during epidemic years. We derived urban landscape classes from the physical and socio-economic factors hypothesized to be associated with West Nile Virus (WNV) transmission and compared those to human cases of illness in 2002 in Chicago and Detroit. The objectives were to improve understanding of human exposure to virus-infected mosquitoes in the urban context, and to assess the degree to which environmental factors found to be important in Chicago were also found in Detroit.

**Results:**

Five urban classes that partitioned the urban space were developed for each city region. The classes had many similarities in the two settings. In both regions, the WNV case rate was considerably higher in the urban class associated with the Inner Suburbs, where 1940–1960 era housing dominates, vegetation cover is moderate, and population density is moderate. The land cover mapping approach played an important role in the successful and consistent classification of the urban areas.

**Conclusion:**

The analysis demonstrates how urban form and past land use decisions can influence transmission of a vector-borne virus. In addition, the results are helpful to develop hypotheses regarding urban landscape features and WNV transmission, they provide a structured method to stratify the urban areas to locate representative field study sites specifically for WNV, and this analysis contributes to the question of how the urban environment affects human health.

## Background

By November, 2006, the West Nile virus (WNV) was associated with 23,593 cases of recorded illness in humans and 905 deaths since its introduction into the United States in 1999 [1]. The primary transmission of WNV to humans is through infected mosquitoes that have fed on infected birds [2]. Between 1999 and 2001, only several dozen human cases were reported each year; however, in 2002, the number of WNV cases in the U.S. increased markedly to 4,156. Illinois and Michigan led the nation in numbers of human cases of illness from WNV that year (884 and 644 cases respectively), with important focal points around Chicago and Detroit. This urban preference and the presence of local foci reflects a similar pattern seen with St. Louis Encephalitis (SLE) in the eastern United States, which exhibits periodic epidemics, often in large metropolitan areas, with some very high local rates [3].

The urban landscape in which the WNV outbreaks occur is a diverse mix of buildings, transportation routes, vegetation, land uses and people. These are associated with many aspects of urban life, from economic activity, to crime, to patterns of illness. These contextual factors are often neglected in research related to disease transmission and public health [4]. In this paper, we analyze the local spatial patterns of the urban landscape in Chicago and their relationship with confirmed infections (i.e., cases) with West Nile virus in people in 2002, and then compare Chicago with Detroit. We do this with a factorial ecology approach in which a principal components analysis defines the dominant trends in the area based on a set of variables and then a cluster analysis recombines those components into relatively homogeneous classes. The goal is to determine the specific landscape characteristics that contributed to their suitability for viral activity during the 2002 outbreak of WNV infections when naïve populations in the Midwestern cities of the United States were first exposed to the virus.

### Urban form, urban metrics and health

Sociologists and geographers have scrutinized urban form, noting especially the land use patterns relative to the Central Business District (CBD). Research in the middle 1900s at the University of Chicago school focused on traditional models of urban form, including concentric rings, based on population growth eras; concentric zones divided into wedges with some long sectors following major transportation routes out from the center of the city; and the urban space as fragmented nuclei serving distinct functions [5,6]. With digital computers and more efficient multivariate statistical analyses, came the urban factorial ecology approach to characterizing cities used, notably, by geographer B.J. L. Berry and colleagues [7]. This approach involved using large numbers of variables to define general patterns through empirical work on numerous cities. More recently, similar research on urban form has focused on spatial metrics that combine many variables to better understand economic activities and land use change [8,9] and landscape characterization [10,11].

Urban form and its relationship with health in Chicago received early attention in a 1939 study by Faris and Dunham [12]. In this seminal work, it was noted that mental disorders in Chicago were associated with more decrepit urban areas and posited that this was due to like individuals living in similar situations and the contribution of physical and social conditions, especially anonymity and high mobility, to mental health. More recently, ecological studies in the United States have considered urban sprawl and increased obesity and the host of chronic diseases that accompanies it [13-15], urban water quality [16], pedestrian-vehicle accidents [17] and mental health [18]. Urban ecologists have also contributed to methods for analyzing urban form and its relationship to ecosystem health [19]. In the realm of natural systems in urban places, Alberti [[20], p 174] writes that " [t]he question of how patterns of human settlements affect ecosystem function is becoming increasingly important in ecology." These issues are particularly important in understanding a vector-borne disease such as WNV illness, where birds and mosquitoes and their interaction with the natural environment in urban places are integral to disease transmission. Alberti's framework for the study of urban ecosystem functions considers the patterns of urban form, heterogeneity, intensity and connectivity, and describes urban form as the degree to which a city is centralized. These same attributes can be considered in terms of urban forms and health.

### Spatial aspects of West Nile virus transmission in urban areas

Infections caused by pathogens by way of a mosquito vector often cluster in space and time given the habitat requirements of the vectors and vertebrate hosts involved in the transmission [21-24]. Examples at all spatial scales, from the local to the continental, provide evidence that vector borne diseases, including malaria and the encephalitis-producing viruses, are related to patterns of climate, vegetation, hydrology, and types of human settlements [25-28].

With WNV, *Culex *species mosquitoes are implicated in transmission in the United States and their feeding habits and habitat requirements are critical to the spatial variability of viral transmission [29-31]. *Culex pipiens *deposit their eggs in stagnant water. Low places with poor drainage, urban catch basins, roadside ditches, sewage treatment lagoons, and manmade containers around houses provide good larval development sites [32,33]. The primary reservoir hosts for WNV are birds, but humans, horses and other mammals can become infected when fed on by an infective mosquito. Virus transmission requires that both mosquito vectors and avian hosts be present in a manner that allows for sufficient and timely interaction among them. Particular bird species and weather conditions are likely important but not certain [34,35]. Recent evidence points to the July dispersion of robins (*Turdus migratorius*) as being associated with the shift among *Culex *mosquitoes from avian to human hosts and the increase in human cases of illness from WNV [36].

In New York City, the pattern of human illness from West Nile virus in 1999 was clustered in space and associated with places where more abundant vegetation was measured using satellite imagery [37]. In Georgia, a state-wide analysis of avian samples revealed increased virus endemicity in birds in urban/suburban areas during the years 2002 to 2004 [38]. In Chicago, areas of high crow mortality were associated with subsequent human cases of WNV in 2002, with the distribution of both events clustered in space and time [39,40]. In addition, human cases in the 2002 Chicago area outbreak were associated with higher percentages of vegetation in census tracts, more post WWII housing, and neighborhoods characterized as relatively wealthy, with a high proportion of census-defined White residents [41].

## Results

### Chicago – with Land Cover of Illinois

In Chicago, 1479 census tracts were included in the classification analysis. Four components were found based on 18 variables in the principal components factor analysis, accounting for 67% of the variance in the original variables. Conceptually, the score coefficients of the four were higher under these conditions:

1. Hilly/natural – low population density, high land cover diversity, high range of elevation, highly vegetated.

2. 70s-80s housing – High percentage of housing built in 1970s and 1980s, with absence of older; but some 1960s housing

3. High income, older, and White – High household income, high percentage of population White, and high population age.

4. 40s-60s housing – Post World War II housing and high percentage of low to medium density urban use.

**Figure 1:**
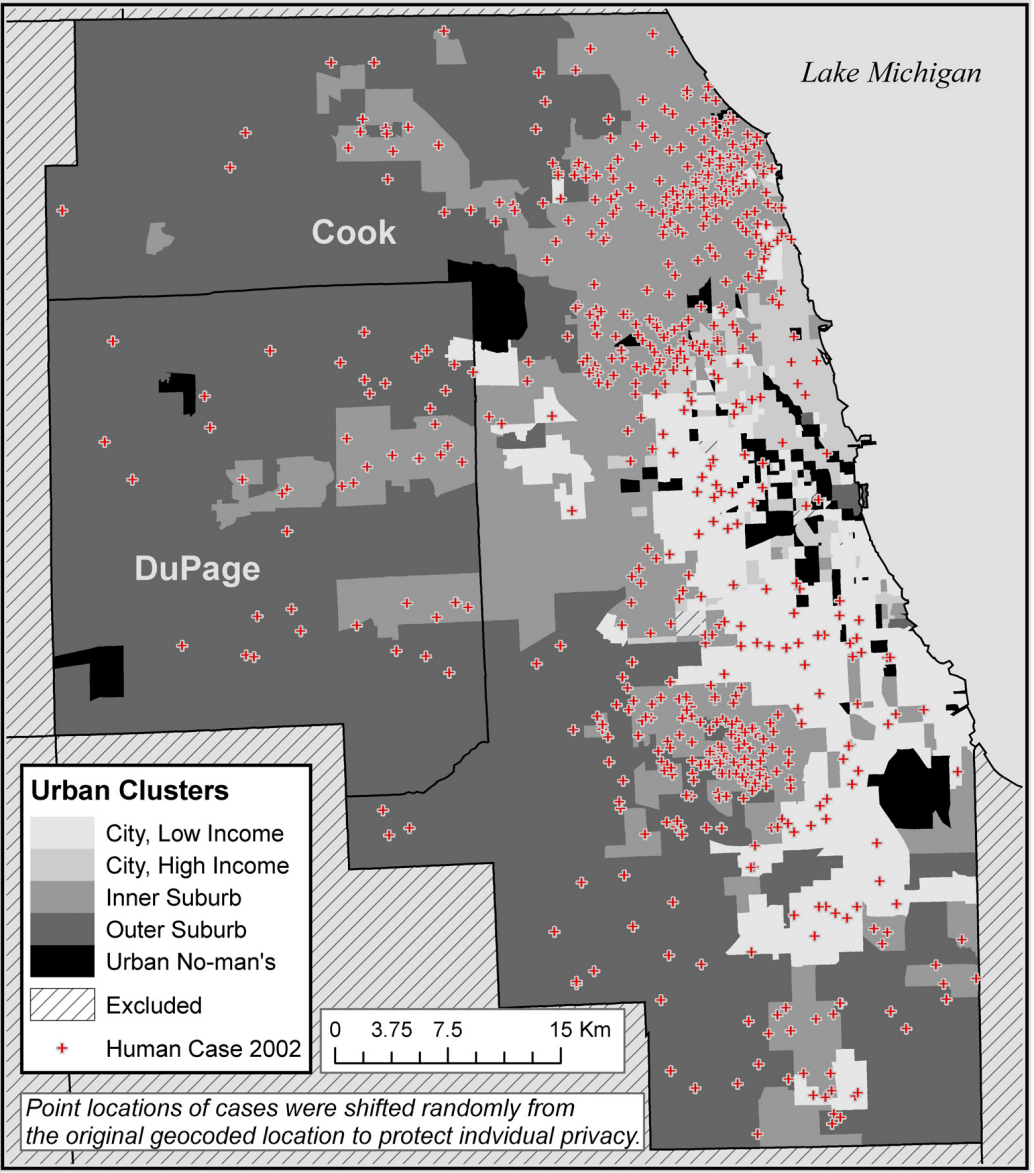
Five urban classes in the Chicago region and WNV illness human cases in 2002.

Five urban landscape classes resulted from the cluster analysis (Figure 1, Table 1). The five classes and their dominant characteristics are as follows:

**Table 1:** Chicago urban classes with WNV human case rates from 2002

Urban Class	Brief Description	Chicago with Land Cover of Illinois				Chicago with National Land Cover Data			
		
		% of tracts	N WNV	Population	WNV per 100 K	% of tracts	N WNV	Population	WNV per 100 K
1	City, Low Income	31.8	99 (15.2%)	1,677,454	5.90	31.9	98 (15.1%)	1,526,551	6.42
2	City, High Income	15.8	25 (3.8%)	894,010	2.80	26.2	132 (20.3)	1,671,026	7.90
3	Inner Suburb	26.2	404 (62.2%)	1,777,327	22.78	26.4	363 (55.8%)	1,895,900	19.15
4	Outer Suburb	19.6	115 (17.6%)	1,819,621	6.32	11.1	50 (7.7%)	1,122,668	4.45
5	Urban No-man's	6.6	7 (1.1%)	112,456	6.22	4.4	7 (1.1%)	64,723	10.82
	OVERALL	100	650 (100.0%)	6,280,868	10.35	100	650 (100.0%)	6,280,868	10.35

1. City, Low Income – young, low percentage White, low income, some 40-60s housing

2. City, High Income – not very natural, high income, low 40-60s housing

3. Inner Suburbs – 40-60s housing, fairly high income, White

4. Outer Suburbs – hilly, natural, low density, higher income

5. Urban No-man's Land – Old housing, natural, airport

In the Chicago area, 685 human West Nile cases were confirmed by the Illinois Department of Public Health in 2002 [42]. Of these, 650 (95%) were successfully geocoded and included in the analysis. The number of cases per 100,000 people was 10.35. In the 376 tracts with at least one case, case counts ranged from one to nine cases.

The incidence of WNV illness varied among the five urban classes. The third class, Inner Suburbs, had 404 of the 650 cases (62.2%) and had the highest incidence of 22.78 per 100,000 people (Table 1). This is more than eight times higher than the lowest rate of 2.80 found in the City, High Income class, with 25 cases. It is more than three times the rate of about 6.00 for the other classes. Analysis of variance tests for differences in tract means further demonstrated differences in WNV incidence among the groups. Using multiple comparisons, the Inner Suburbs class was significantly different (p=.01) than each of the other groups and the *a priori *contrasts option in SPSS with a t statistic revealed a difference (p=.01) in the Inner Suburb group and the other groups combined. Visits to the five urban classes in Chicago confirmed the dominant features as predicted by the cluster analysis classes.

The detailed assessment of a 5% random subset of tracts (N = 78) revealed that the subset tracts in the first and fifth classes were all correctly classified. The second, third and fourth classes had three, five, and two tracts misclassified respectively. Overall, about 78% of the tracts examined were correctly classified.

### Detroit

From the three-county area in Michigan, 1084 tracts were included after excluding very rural tracts. The same 18 variables were used as above, with the exception that the National Land Cover Dataset (NLCD) was used instead of the Land Cover of Illinois (LCOI) data, for which there was no Michigan equivalent. Four components were found that accounted for about 73% of the variance in the original variables. As in Chicago, two of the components were focused on housing age, one measured natural features and terrain, particularly hilliness and vegetation and the fourth component measured socio-economic factors.

**Figure 2:**
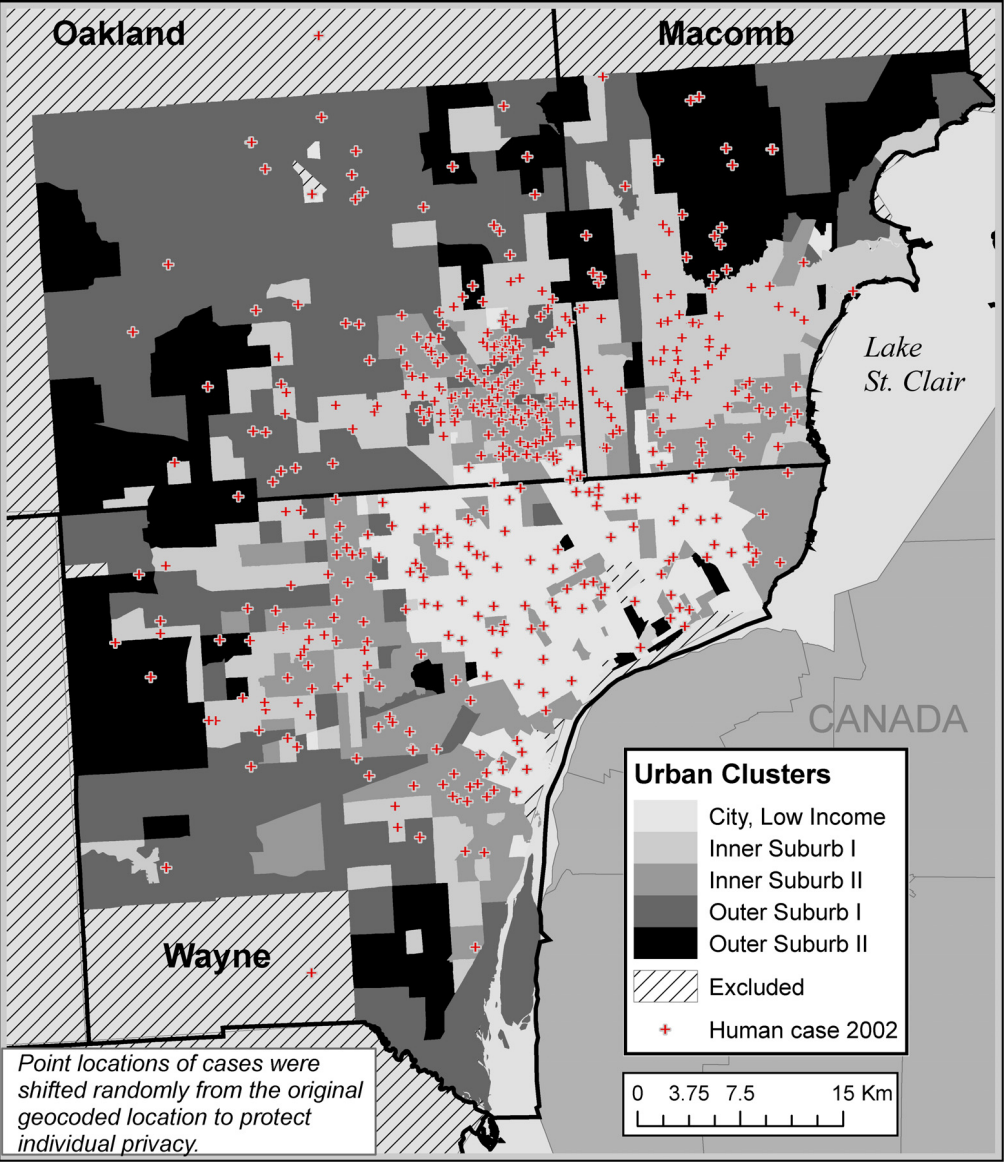
Five urban classes in the Detroit region and WNV illness human cases in 2002.

In Detroit, five urban landscape classes resulted from the cluster analysis (Figure 2, Table 2). The five classes and their dominant characteristics were

**Table 2:** Detroit urban classes with WNV human case rates from 2002

Urban Class	Brief Description	Detroit with National Land Cover Data			
		
		% of tracts	N WNV	Population	WNV per 100 K
1	City, Low Income	27.3%	97 (19.7%)	967,645	10.02
2	Inner suburbs I – 60s Suburbs	19.7%	124 (25.2%)	806,614	15.37
3	Inner Suburb II – 40s-50s	23.7%	187 (37.9%)	821,700	22.76
4	Outer suburbs I	11.2%	30 (6.1%)	506,780	5.92
5	Outer suburbs II	18.0%	55 (11.2%)	650,756	8.45
	OVERALL	100	493 (100.0%)	3,753,495	13.13

1. City, Low Income – Low income, high percentage Black, high density population, low vegetation, flat

2. Inner Suburbs I – Housing from 1960s or newer, older, White residents

3. Inner Suburbs II – Housing from the 40s and 50s with White residents. Flatter, less vegetation and lower elevation than Inner Suburbs I.

4. Outer Suburbs I – New housing, an absence of post WWII housing, high income

5. Outer Suburbs II – Very natural, some 60s housing, high land cover diversity. Lower housing density and less hilly than Outer Suburbs I.

For 2002, 525 human cases of illness from WNV were confirmed by the Michigan Department of Community Health for the three counties of Macomb, Oakland and Wayne. Of these, 501 cases (94%) were successfully geocoded from addresses. The 493 cases that were located in the tracts of interest indicated an incidence of 13.13 per 100,000 (Table 2). Tracts with at least one case had from one to seven cases.

As with Chicago, the class with 1940s and 50s housing (Inner Suburb II) had the highest incidence of WNV infection (22.76 per 100,000). The second highest rate was in the Inner Suburb I class. The lowest rates were in the two Outer Suburbs classes (5.92 and 8.45 per 100,000 people). The fifth class, City, Low Income, had a moderate rate at 10.02, about one half of the highest rate

The detailed assessment of a 5% random subset of tracts (N = 43) revealed that the first and fifth classes were all correctly classified. The second, third and fourth classes had two, one, and one tracts misclassified respectively. Overall, about 90% of the tracts examined were correctly classified.

### Chicago – with National Land Cover Dataset

When the Chicago analysis was repeated using the NLCD data in place of the LCOI, the results were similar to the first Chicago analysis. The four factors are conceptually the same as for the other Chicago results. The components accounted for about 67% of the variance in the original variables. Several differences in the outcome of the principal components analysis are noted, with the percentage of a tract that is low density urban loading on the hilliness/natural factor in the NLCD analysis, while the most comparable value of low to medium density urban for the LCOI loaded on the 40s-60s housing.

In terms of the clusters; again, the clusters are similar, with essentially the same conceptual basis as in the original Chicago analysis (Table 1). The Inner Suburb class again has the highest rate of the NLCD classes at 19.15 per 100,000 people. The most important difference occurs with the class of City High Income when using the NLCD. It is both larger in extent than when LCOI is used and it encompasses many more of the WNV human cases than in the original set of classes. It has 132 cases with a rate of 7.90 per 100,000 compared to the original values of 25 cases and 2.80 cases per 100,000. Although the absolute difference in means is not as notable as with the LCOI results, the mean of tract rates in the Inner Suburb class was significantly different (p < .01) from three of the four other classes. The exception was the class incidence for Urban No-man's which was close to the overall incidence of WNV illness, and not significantly different from any of the other classes. The Inner Suburb class was also significantly different from the three classes, excluding Urban No-man's, when these were tested as a group.

The detailed assessment of a 5% random subset of tracts (N = 66) revealed that the fifth class was correctly classified. The first, second, third and fourth classes had two, five, three and one tracts misclassified respectively. Overall, about 60% of the tracts examined were correctly classified.

## Discussion

The urban landscape characterization in Chicago and Detroit marked similarities in the two areas, reflecting their common history of growth. This growth was typified by strong early development during the late 1800s followed by a period of steady but moderate growth and then a surge post World War II. The distinctive patterns of age of housing and the concomitant vegetation and socio-economic features in places with similar growth patterns were reflected in both the initial components and the urban classes derived from the cluster analysis. In both cities, but more obviously in Chicago, the concentric rings of urban growth are evident in the urban class pattern. The areas that are more natural and would provide habitat for mosquitoes are more common outside the CBD, but the degree to which an area is natural is not a simple gradient measured as a transect out from the CBD. The wedges along early transportation routes can be seen in the Urban No-man's class in the relatively natural aspect of large industrial areas. In addition, urban centers based on both older and newer commercial activity are located throughout the urban area. These patterns illustrate the notion of the landscape as palimpsest, where past use and intentions leave an imprint that remains visible and influential today; in this case, providing suitable mosquito habitat and the transmission of WNV to humans.

Overall, the urban classes that result from the NLCD analysis available for both cities were not as successful in identifying WNV transmission regimes as was the LCOI-based analysis outcome, available only for Chicago. Notably, in the Chicago area, the City, High Income class with NLCD included WNV cases that were in the Inner Suburb class with the LCOI. This difference may have occurred because NLCD's urban land cover classes did not distinguish between high and low density commercial and industrial use. The LCOI was thus better at making a distinction related to the amount of more natural open space and impervious surface, a distinction that would be more helpful for identification of potential mosquito habitat. On the other hand, the classification accuracy was highest for the Detroit classes. This illustrates a need for a dynamic view of urban form, where the manner in which it is measured needs to be fitted to the problem at hand.

Housing age is both a dominant feature of the urban structure and is strongly associated with WNV in 2002. This warrants further investigation. It is possible that the storm water sewer system that dates from this time accounts for the relationship, since water drainage and the degree to which standing water combines with organic matter affects breeding conditions for *Culex *mosquitoes, especially in times of drought when the water stands for a long period of time [34]. The vegetation regime of the Inner Suburbs is also of interest. These areas have a distinctive set of vegetation, including 50–60 year old hardwood trees, small yards with shrubs, grassy alleys, railroad rights of way, and cemeteries. The productivity and species richness of these small natural areas in highly urban spaces may hold a key to understanding WNV amplification and transmission.

The Outer Suburbs, with more vegetation and high diversity of land cover than other areas had fewer cases and lower rates of WNV. Given the tendency for native flora and fauna as well as biodiversity to be higher in those outer suburbs [20], lower biodiversity may be associated with WNV transmission. High risk areas may have sufficient vegetation and other characteristics that allow for the combination of birds and mosquitoes needed to sustain transmission. The role of robins in this particular environment may be of special importance. If the dispersal of robins is related to the increase in WNV illness in humans as suggested by Kilpatrick et al. [36], then why is this effect more important in some urban neighborhoods than in others? This question should be explored more fully through more precise measurements of mosquito productivity, avian species availability and the intensity of WNV infection in mosquitoes and birds.

Additional socio-economic and land cover variables may be helpful. The percentage of residents that were "White" was a strong variable in the statistical analysis. Given the possible importance of ethnicity in this analysis, future analysis might include Hispanic or Asian population distributions. The possible importance of impervious surfaces indicates the need for direct use of satellite imagery and knowledge to soils characteristics to determine more precisely the patterns of impervious surfaces rather than inferring that from existing land cover maps. Mosquito infections are a necessary precursor to human infections and the question remains as to whether the association of mosquito infection spatial variability follows the same patterns as those seen in human illness. Precipitation differences across the urban areas may provide additional clues to the patterns observed and also bring in the need for temporal analysis. These phenomena may need to be measured at a different scale than the current analysis, with smaller regularly shaped units being more appropriate for the heterogeneous natural phenomena or different administratively based units suitable for socio-economic measures. Given the importance of hydrology to the life cycle of mosquitoes, geographic units based on watersheds or catch basins should be considered.

## Conclusion

The urban classes developed from the combination of natural and socio-economic features in Chicago and Detroit indicate a relationship between WNV transmission risk and the age of housing, land use, and the concomitant social and natural features. In addition to the development of hypotheses related to WNV transmission in the urban Midwest, the landscape classes provide a succinct and quantitative approach to selection of field sites for mosquito and avian collection and observation. This will be done through stratification using the urban classes derived here as important dimensions of the urban landscape relative to this vector-borne disease. The comparison of the results from Detroit and Chicago indicate that the associations may be generalized and have implications for other cities. The contrast between results with LCOI and NLCD land cover classes provide background information on which to base future efforts to measure vegetation and imperviousness to better understand the risk associated with these factors.

The absolute location of the tracts assigned to urban classes is not as important as the characteristics of the neighborhoods found in those classes. At a different spatial scale, the same characteristics may be found in other parts of the urban space though that tract did not fall into a particular class in a statistical sense. In 2002, the first year of a significant WNV outbreak when host populations were relatively naïve, high rates of the illness indicate a particularly high vulnerability relative to other places not just to the hosts but also to the neighborhoods in which they reside. West Nile virus activity was low for two years following the 2002 outbreak but the two subsequent years, 2005 and 2006, saw a significant increase in illness in both Illinois and Michigan [1]. Human illness from West Nile virus is an ongoing problem and the results of this analysis will help to identify the most vulnerable neighborhoods and will be useful in the future investigation of transmission of the WNV in similar urban areas.

## Methods

The approach used here is influenced by urban factorial ecology studies developed in the 1960s in which principal components factor analysis characterizes urban areas by deriving uncorrelated components from a set of variables that reflect important characteristics of a neighborhood [7,43]. The variables used were those that measure natural and anthropogenic landscape characteristics conducive to the transmission of the WNV. The components then were used to derive homogeneous classes, which represent urban form. Similar classification methods are used by market analysts and for classification of remotely sensed imagery. Market analysts classify neighborhoods from census data, and provide descriptive names such as "Blueblood Estates" or "Norma-Rae Ville" [44,45]. In remote sensing, features are recorded as digital numbers measured using electromagnetic sensors and the spectral signature of features leads to land cover classes such as "Shrubland" or "Low Intensity Residential" [46]. With geographic information processing and multivariate statistics, we use both natural environment variables, including vegetation and topography in combination with housing age, population density and socio-demographic factors to characterize urban neighborhoods and then assess how effectively the neighborhoods support the transmission of WNV.

The Great Lakes cities of Chicago and Detroit are the study sites, and both are at about the same latitude around 42 degrees north. They have similar geological features formed during the retreat of massive ice fields about 14,000 years ago [47], with relatively flat terrain on clay soils cut by drainage channels with sandier soils. These areas are prone to flooding and standing water. The Chicago study area comprises the two counties of DuPage and Cook in the state of Illinois. The Detroit, Michigan, study area is located in Macomb, Oakland and Wayne County, Michigan.

The United States census tract is the unit of analysis. The census data play an integral part in the analysis and prior exploration of the data indicated that tracts captured the regional differences in WNV disease incidence during 2002 without being redundant [41]. That is, this scale was judged to be a good match to the properties and processes of interest. Data on the demographic and natural characteristics of the region were summarized as needed to bring those measures into the census tract unit using the ESRI ArcGIS 9.1 software (Redlands, CA).

West Nile illness human case data are from the Illinois Department of Public Health and the Michigan Department of Community Health. Point locations of cases were generated using address geocoding with ESRI Streetmap USA (Redland, CA). Those addresses that could not be geocoded were located manually using electronic and paper maps. In both Illinois and Michigan, cases included both the more severe, neuroinvasive disease and milder forms of illness.

The socio-economic and housing variables included in the analysis were: the percentage of tract population that is White, median household income, median age of the population, average age of housing, percentage of housing that was built in each decade from 1940 to 2000, and percentage of housing built before 1940. These data are from the Census Bureau 2000 Census of Population and Housing [48]. Population density was measured both as density of people and as density of housing units in a tract.

Other environmental variables included vegetation, land cover and topography. Vegetation and land cover variables for each tract were derived from the Land Cover of Illinois (LCOI) data [49]. For Michigan the National Land Cover Data of 1992 (NLCD) was used [50]. With both datasets, the original classes were reclassified as either "vegetated" or "not vegetated" and percentage of tract vegetated calculated. Vegetated status was based on the degree that impervious surfaces were present, since impervious surfaces are a good indicator of urban land use [51]. Land cover diversity was developed as the number of different land cover classes found in a tract. A third land cover variable was the percentage of low to medium density residential area. For terrain, average elevation above mean sea level and the range of elevation were calculated from a 30 meter digital elevation model [52]. With both the land cover and elevation data, the original data were in a grid format that was summarized to conform to the census tract polygons.

This set of 18 correlated variables was reduced to uncorrelated components using the factor analysis principal components method in SPSS version 12.0 (Chicago, IL) [53,54]. The number of components retained was determined through examination of the slope of the skree plots and the eigenvalue output, which show the additional amount of variance explained by additional components. The eigenvalues describe the amount of variance explained by that component. The goal in selecting the number of components was to account for as much variance as possible with the fewest meaningful components. A varimax rotation aided interpretation of the components. Finally, factor score coefficients were saved as new variables using a regression method.

The factor score coefficients were used as the input variables in a cluster analysis. The output of the cluster analysis is a set of classes with tracts with similar characteristics placed in the same class [55,56]. For the cluster analysis, a two-step method was used. First, a features tree was constructed, such that each tract was placed in a new node or in an existing node based on its similarity with other nodes. Then, the nodes were grouped using an agglomerative clustering algorithm. These are compared to statistical criteria to determine the number of clusters to create. Parsimony was achieved with a small number of clearly defined classes. Since the Detroit and Chicago land cover datasets were different, one additional landscape classification was performed for Chicago, using the NLCD in that area.

The raw incidence rate of West Nile virus human cases (number per 100,000 people) in a tract provided evidence of the class' suitability for WNV transmission. As an additional assessment of the difference in the degree to which WNV illness affected the different urban classes, we used an analysis of variance (ANOVA) on the tract means of the incidence of WNV for the different urban classes [55]. The Levene statistic test results revealed that the variances were not homogeneous, so the Welch and Brown-Forsythe ANOVA tests used, where homogeneity was not assumed. Additional ANOVA options were employed whereby post-hoc tests (Tamhane method) considered differences in means among the various pairs of urban classes and between one class and a group of all others.

After the urban classes for each city were developed, we determined the degree to which the urban class definitions were in accordance with observed tract characteristics. This was done for a 5% sample of tracts. For this assessment, tract characteristics based on the original set of 18 variables were measured for all tracts and then compared to the random set of tracts from each of the five classes. The variables considered were those represented by the dominant features of each class as described above. When most of the features observed in the subset of tracts were representative of its class compared to the overall mean of the key variables, then that tract was considered correctly classified. When two or more important characteristics were not consistent with the class, the tract was considered to be incorrectly classified.

## Abbreviations

ANOVA – Analysis of variance

CBD – Central Business District

LCOI – Land Cover of Illinois

NLCD – National Land Cover Dataset

SLE – St. Louis Encephalitis

WNV – West Nile virus

## Competing interests

The author(s) declare that they have no competing interests.

## Authors' contributions

MR conceived and conducted the analysis and wrote the original draft of the paper. EW provided expertise on mosquito habitat in urban environments and contributed to the writing of the paper. EF created the WNV data for Detroit and supplemented the interpretation of the results for Detroit. LH helped provide access to the Illinois WNV data and contributed to the interpretation of results for Chicago. UK contributed to the analysis interpretation and to the writing of the final paper.
